# Supplementary material for: Higher gametocyte production and mosquito infectivity in chronic compared to incident Plasmodium falciparum infections
Source: Nat Commun. 2021 Apr 26;12:2443. doi: 10.1038/s41467-021-22573-7 (PMC8076179; doi:10.1038/s41467-021-22573-7)
Supplement: Supplementary file 1 — Supplementary Information [file 41467_2021_22573_MOESM1_ESM.docx]

**Supplementary Information**

Belonging to: Higher gametocyte production and mosquito infectivity in chronic compared to incident *Plasmodium falciparum* infections. Barry, Bradley, Stone *et al*.

**Supplementary figures**

**Supplementary figure 1: Study visits for participants.** White filled circles are weekly (incident infections only) or monthly (chronic infections only) screening visits. Black filled circles are at day 0 on the x-axis, and represent the point of full enrolment (confirmation of incident infection, or confirmation of chronicity). Grey filled circles are intensive follow-up sampling points. Red dots are visits with clinical symptoms and black crosses are visits where a mosquito feeding assay was conducted. Individuals are ordered by the duration of screening prior to enrolment. **A.** Incident infection sub-study, individuals who remained asymptomatic. **B.** Incident infection sub-study, individuals who became symptomatic during follow-up. **C.** Chronic infection sub-study, individuals who remained asymptomatic. **D.** Chronic infection sub-study, individuals who became symptomatic during follow up.


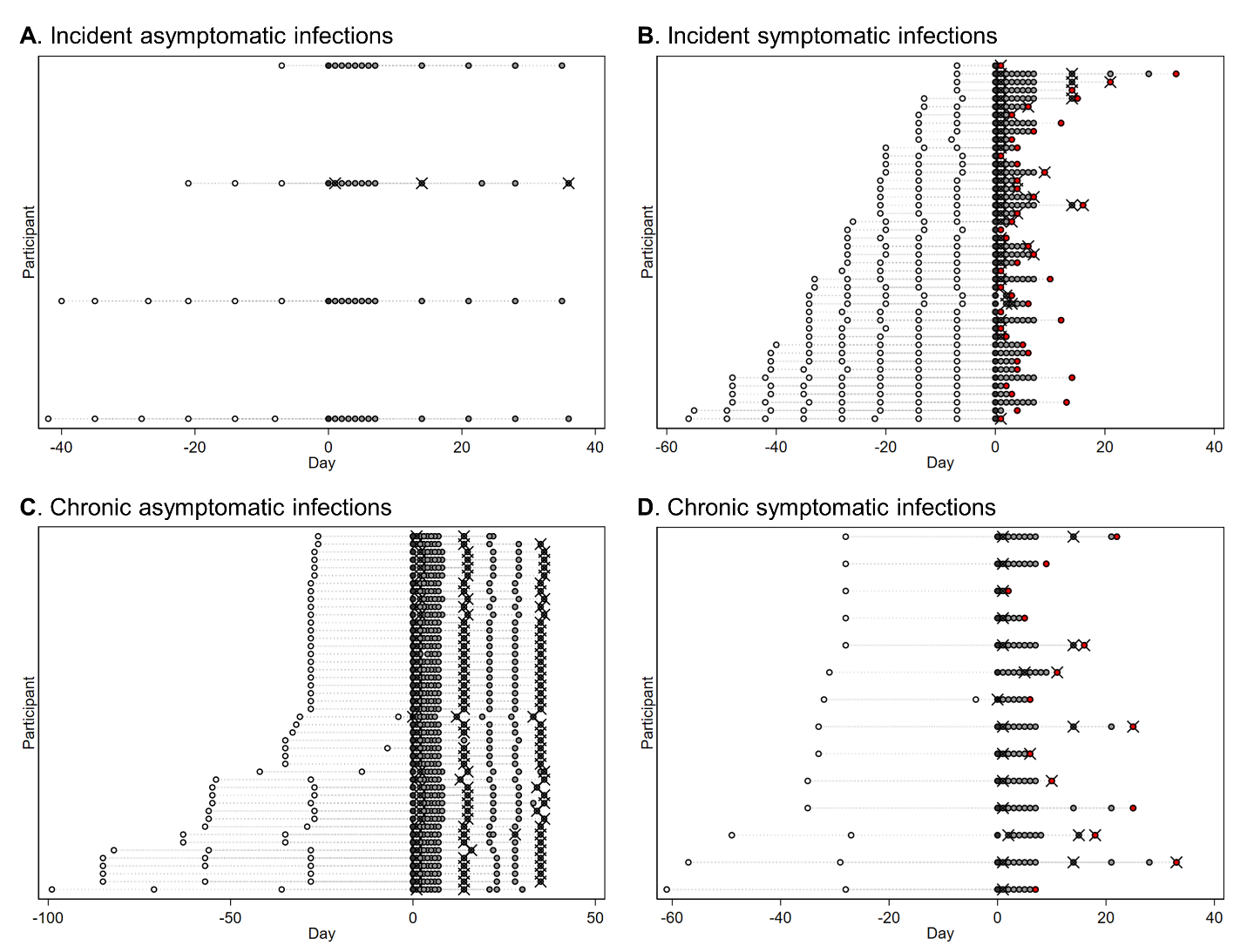


**Supplementary figure 2: Infection trajectories.** Black lines are total parasite density (parasites/µL) as determined by 18s qPCR. Red lines are female gametocyte trajectories determined by Pfs25 based qRT-PCR (female gametocytes/µL), and blue lines are male gametocyte densities (male gametocytes/µL) as determined by PfMGET based on separate qRT-PCRs. Red dots indicate the occurrence of a clinical malaria episode. Three individuals became symptomatic at time points without concurrent parasite density measures, as indicated by red asterisks; *Clinical on day 15, last observation with density day 14, **Clinical on day 16, last observation with density day 14, ***Clinical on day 25, last observation with density day 21.**A.** Incident infections. **B.** Chronic infections.


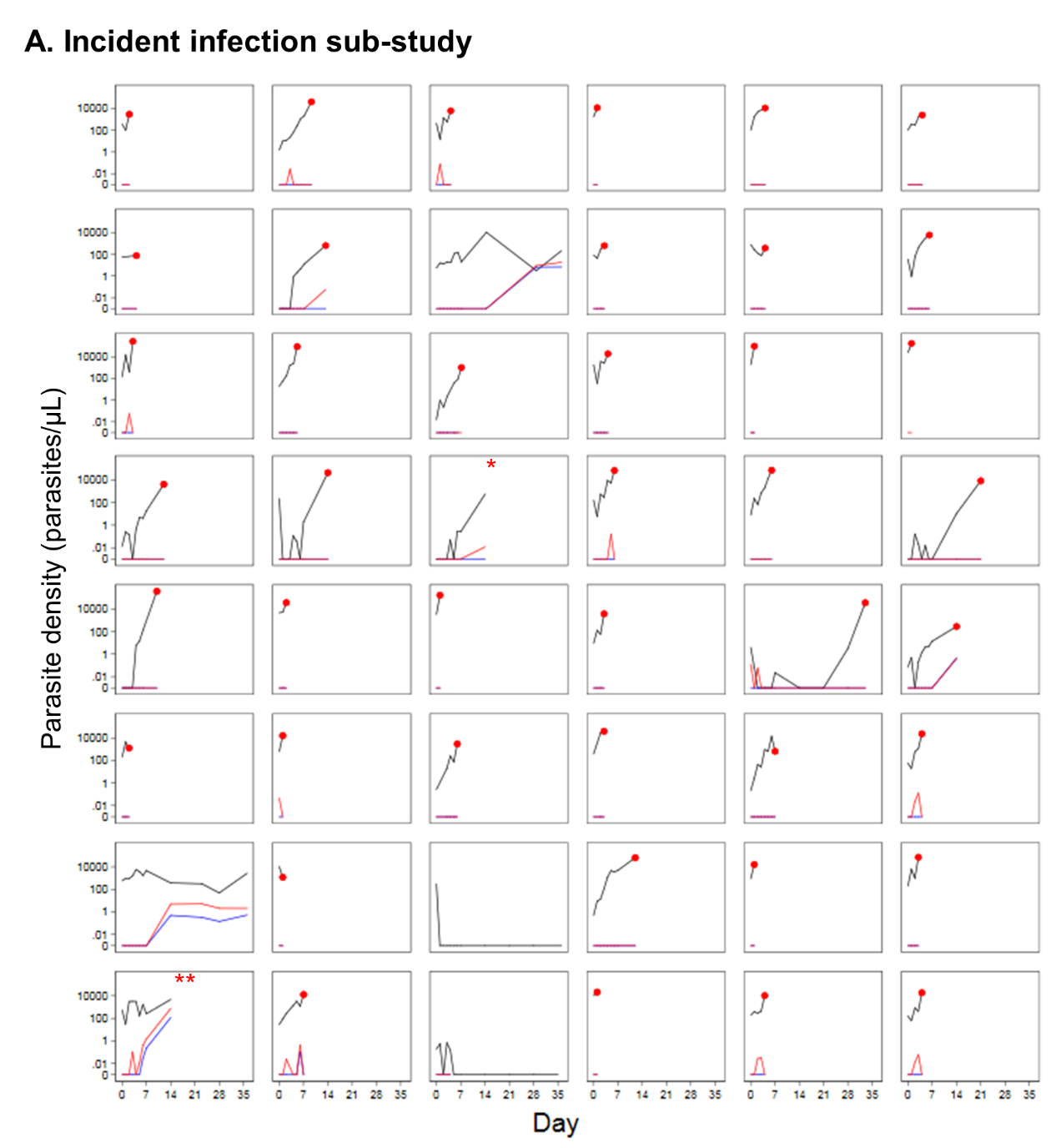


**
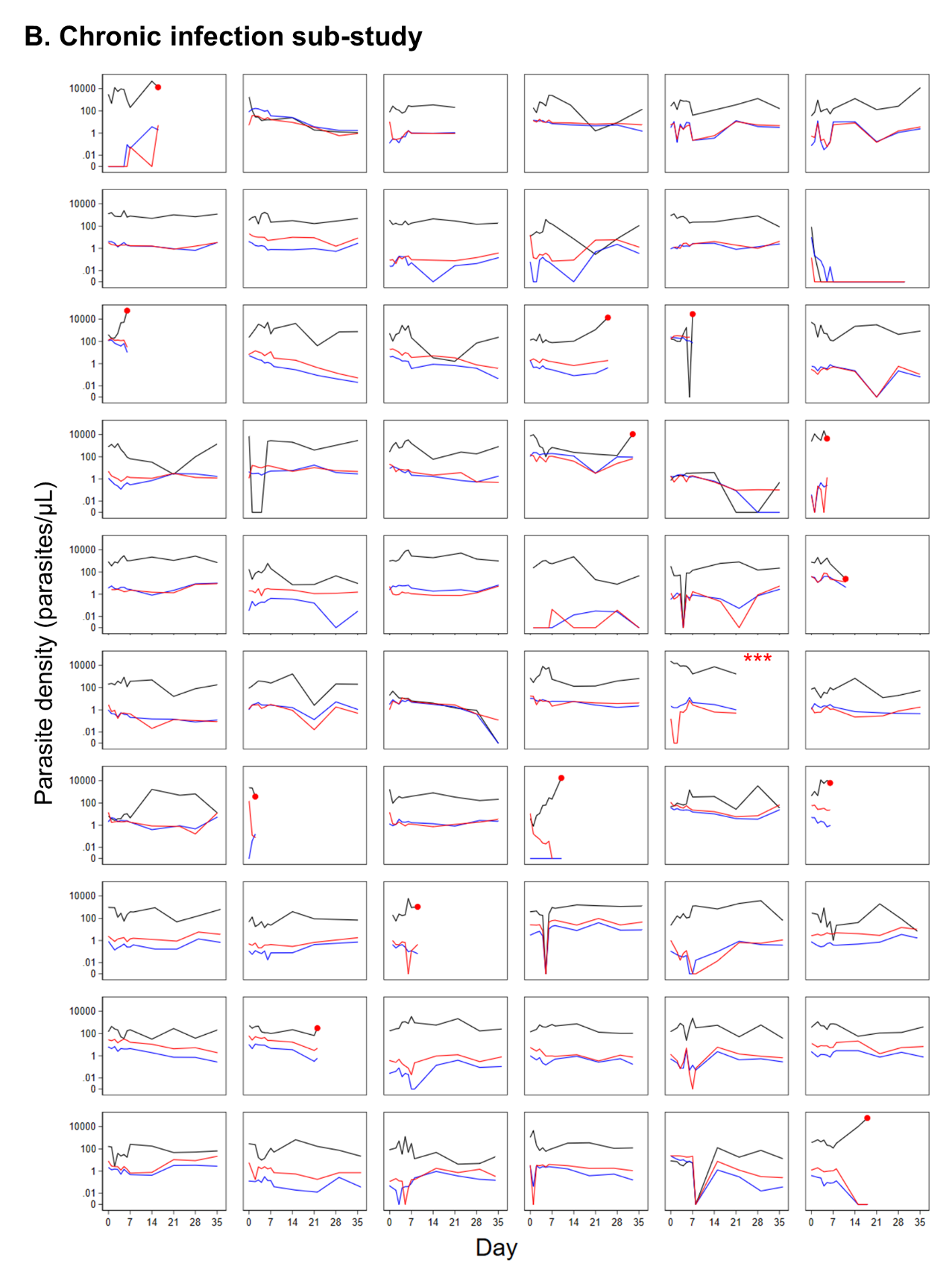
**

**Supplementary figure 3: Gametocyte density change and association with total parasite density.**

**A.** Change in gametocyte density over 48-hour intervals in the first 7 days of follow-up. 294 measurements of gametocyte density change from 56 participants where available (median per person 6, IQR [5,6]). Chronic asymptomatic: 235 observations (44 individuals), median observations = 6, IQR (4.5,6); Chronic symptomatic: 59 observations (12 individuals), median observations = 5.5, IQR (5,6). The dotted line at value 1 indicates no change in asexual/gametocyte density. Black crosses indicate the geometric mean density change multiple for each individual. Overall, gametocyte densities slightly decreased in the first week of observation (mean change over 48hr intervals = 0.87 [95% CI 0.77-0.97]). **B.** The association of gametocyte density and total parasite density at day 14 (combined Spearman correlation -0.19 p=0.1292); the association of gametocyte densities at day 14 were significantly stronger with total parasite densities at day 0 (Spearman rho 0.48 p=0.0001).

**
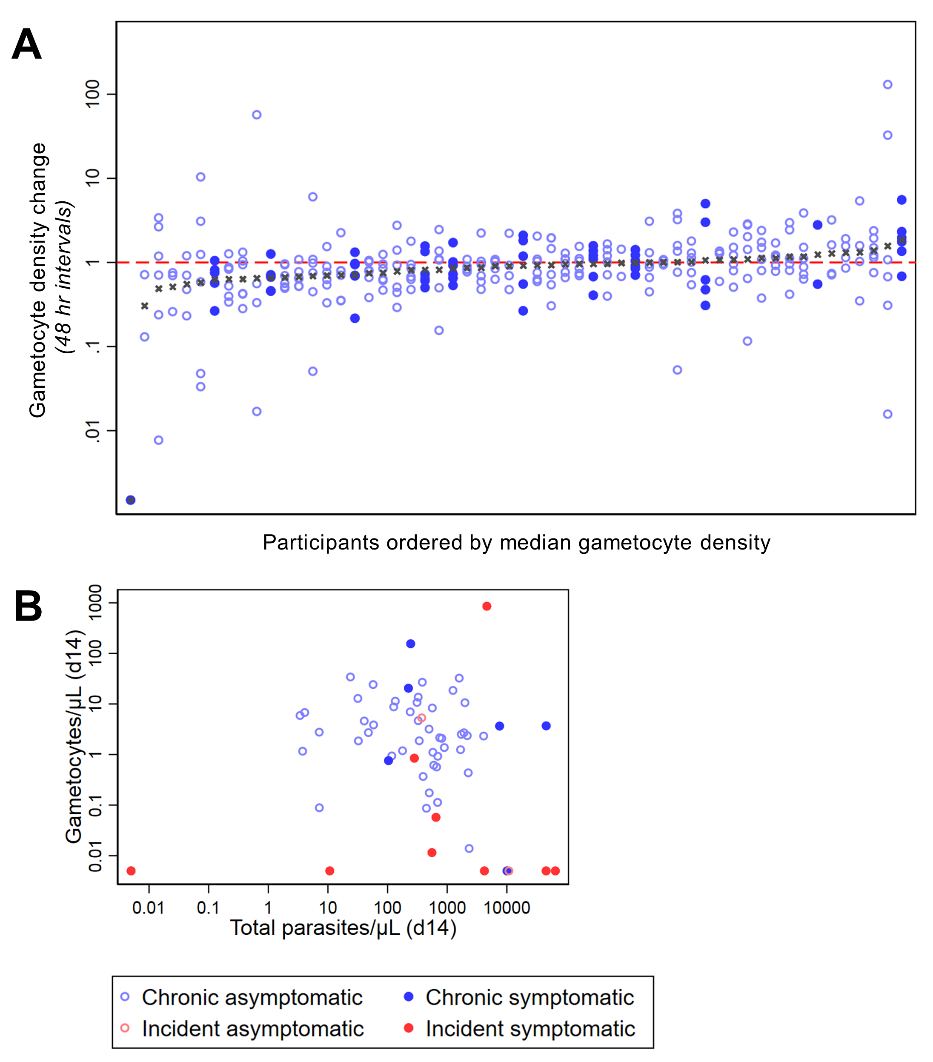
**

**Supplementary figure 4: Gametocyte sex ratio and anaemia. A.** Percentage of total gametocytes that are male. This figure is based in 11 observations from incident (acute) infections, 436 observations from chronic asymptomatic infections and 85 observations from chronic symptomatic infections. Box plot boxes span the median, 25th and 75th percentiles; box plot whiskers span the adjacent values with outliers shown as dots. **B.** Percentage of gametocytes that are male at day 14 after enrolment against haemoglobin level (g/dL) at enrolment. Gametocyte sex ratio (the percentage of the total that were male) was significantly higher in chronic infections (median 38.1%) than incident infections (20.4%) (p=0.027). Sex ratio did not differ between chronic infections that became symptomatic and those that remained asymptomatic throughout follow up (p=0.606). There was no association between Hb density at enrolment and gametocyte sex ratio at day 14 for participants with incident infections (p=0.482), chronic asymptomatic infections (p=0.620), or chronic infections which became symptomatic (p=0.116).

**
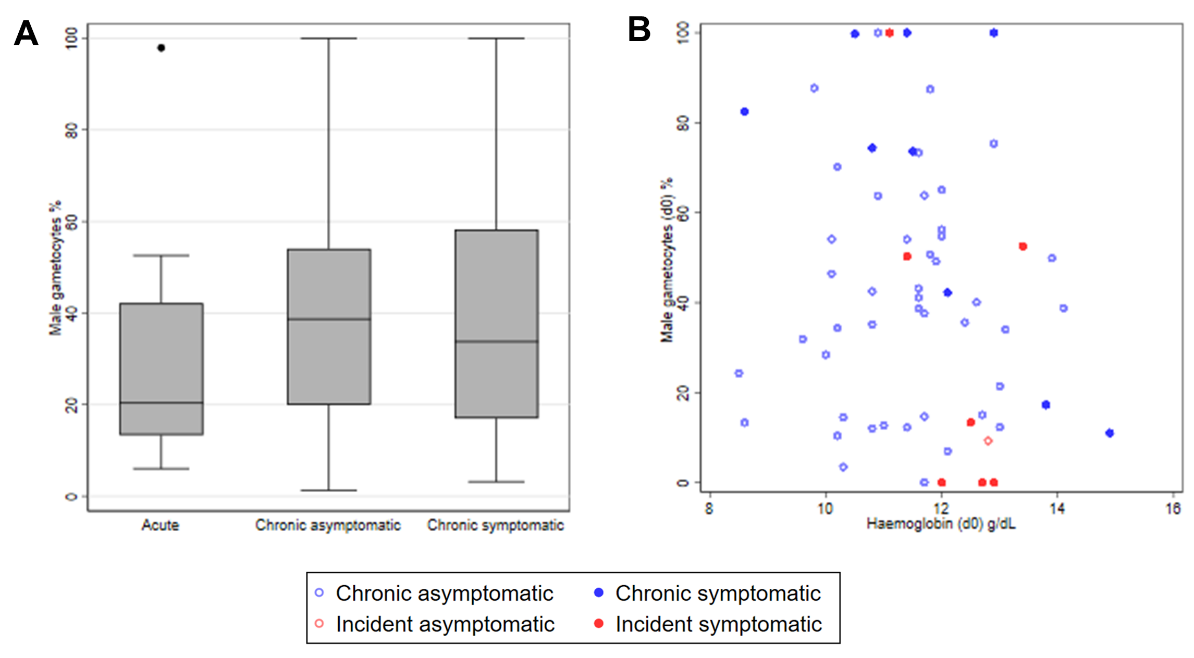
**

**Supplementary figure 5: The association of mosquito infectivity and total parasite density.** There was no significant correlation between total parasite density and infectivity (Spearman correlation 0.12, p=0.081). Diagnostic thresholds are approximations based on the available literature (Kobayashi 2015. DOI: <https://doi.org/10.4269/ajtmh.15-0004>; Snounou 1993. DOI: <https://doi.org/10.1016/0166-6851(93)90077-B>).


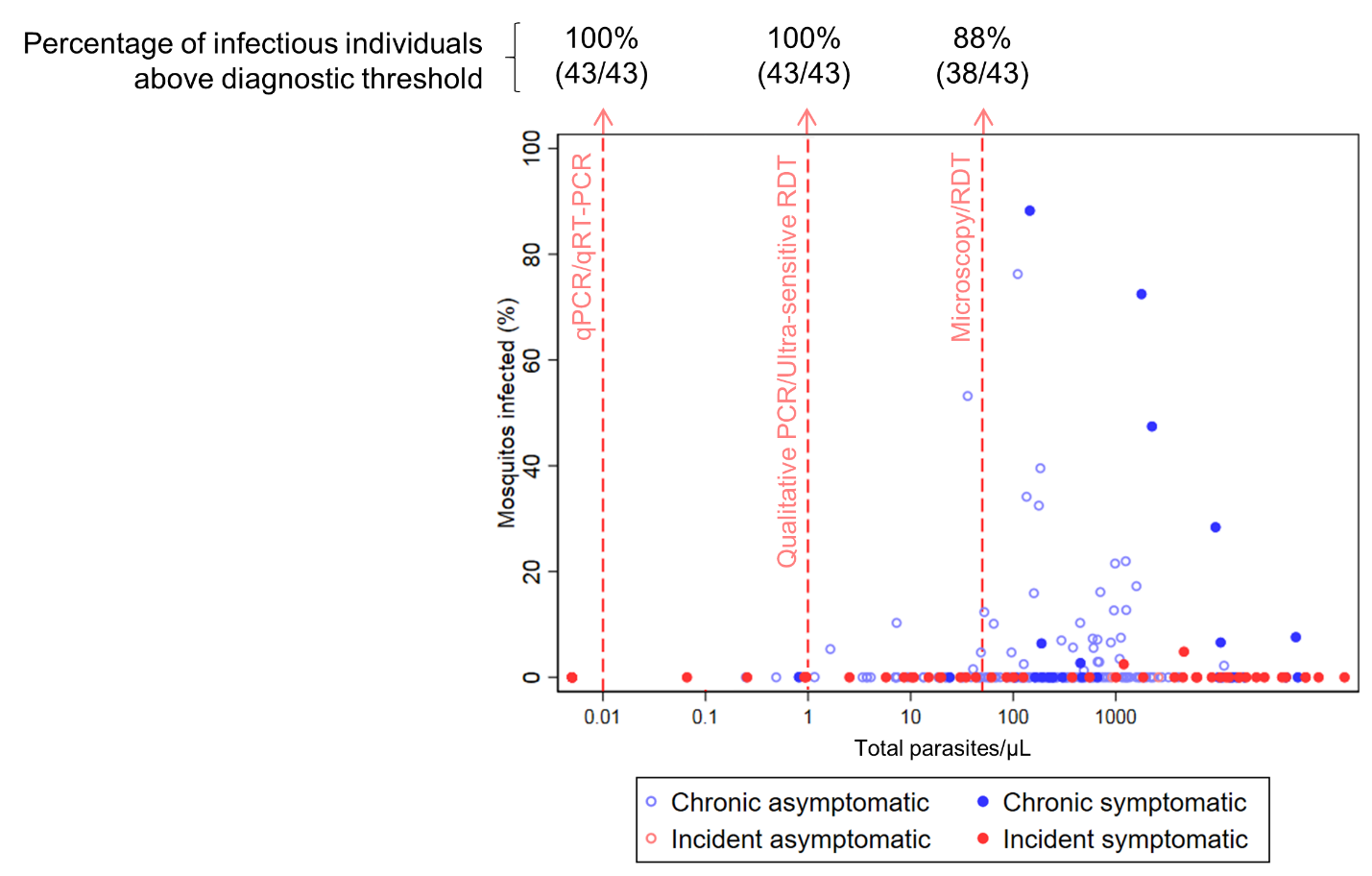


| **Gene ID** | **Acronym** | **Description** | **Location** | **Expression tag** | **Strain** | **Reference** |
| --- | --- | --- | --- | --- | --- | --- |
| PF3D7_0304600 | CSP | Most predominant and antigenic protein on sporozoite surface. Component of RTS,S vaccine | Sporozoite | n/a | 3D7 | [1] |
| PF3D7_1301600 | EBA140 RIII-V | erythrocyte binding antigen 140; involved in erythrocyte invasion | Apical organelles, micronemes | GST | 3D7 | [2] |
| PF3D7_0731500 | EBA175RII_F2 | erythrocyte binding antigen 175; RBC binding region via glycophorin A | Apical tip | GST | 3D7 | [2] |
| PF3D7_0102500 | EBA181 RIII-V | erythrocyte binding antigen 181; involved in erythrocyte invasion | Apical tip | GST | 3D7 | [2] |
| PF3D7_1035300 | GLURP R2 | Glutamate rich protein R2 | Merozoite Surface | n/a | F32 | [3] |
| PF3D7_0206800 | MSP2 CH150/9 | CH150/9 allele of MSP2. Full-length. | Merozoite surface | GST | CH150/9 | [4] |
| PF3D7_0206800 | MSP2 Dd2 | Dd2 allele of MSP2. Full-length. | Merozoite surface | GST | DD2 | [5] |
| PF3D7_1133400 | AMA1 | Apical membrane antigen 1 | Micronemes | His | FVO | [6] |
| PF3D7_0930300 | MSP1-19 | 19kDa fragment of MSP1 molecule. | Merozoite surface | GST | Wellcome | [7] |
| PF3D7_1335400 | Rh2_2030 | Reticulocyte-binding protein homolog 2; involved in erythrocyte invasion | Merozoites; Rhoptries | GST | 3D7 | [8] |
| PF3D7_0424200 | Rh4.2 | Reticulocyte-binding protein homolog 4; involved in erythrocyte invasion | Merozoites; Rhoptries | GST | 3D7 | [9] |
| PF3D7_0424100 | Rh5.1 | Reticulocyte-binding protein homolog 5; involved in erythrocyte invasion | Merozoites; Rhoptries | His | 3D7 | [10] |
| PF3D7_1346700 | Pfs48/45-10c | Gametocyte/gamete surface protein, 10c fragment | Mature gametocytes/gametes | His | 3D7 | [11] |
| PF3D7_0209000 | Pfs230-CMB | Gametocyte/gamete surface protein, CMB fragment | Mature gametocytes/gametes | His | 3D7 | [12] |

**Supplementary table 1:** Characteristics of antigens included in the multiplex bead-based antibody density assay

**Supplementary table 2: Association of serological measures with total parasite multiplication rate (PMR) and symptomatic status.** Spearman correlations were between MPMR (geometric mean of all observations, as described in figure 3) and antibody density (background adjusted median fluorescence intensity [MFI]). Antibody density was assessed at the onset of incident infections (day 0), or at the confirmation of chronic infection (day 0). Wilcoxon rank-sum tests were used to test the difference in net MFI between individuals who remained asymptomatic and those who developed symptoms during follow-up. P-values are for 2-sided tests.

|  | | **Incident infection** | | | | | | **Chronic infection** | | | | | | **Difference between cohorts** |
| --- | --- | --- | --- | --- | --- | --- | --- | --- | --- | --- | --- | --- | --- | --- |
|  |  | **Correlation of MFI with PMR** | | | **Difference in MFI with symptoms** | | | **Correlation of MFI with PMR** | | | **Difference in MFI with symptoms** | | |  |
|  |  | **R^2^** | **p** | **N (total)** | **p** | **n (symptomatic)** | **N (total)** | **R^2^** | **p** | **N (total)** | **p** | **n (symptomatic)** | **N (total)** | **p** |
| **Antigen** | **CSP** | -0.25 | 0.159 | 34 | 0.011 | 44 | 48 | -0.048 | 0.745 | 49 | 0.353 | 13 | 51 | <0.0000001 |
|  | **EBA140** | -0.12 | 0.506 | 34 | 0.576 | 44 | 48 | 0.036 | 0.804 | 49 | 0.060 | 13 | 51 | <0.0000001 |
|  | **EBA175** | -0.27 | 0.127 | 34 | 0.765 | 44 | 48 | 0.015 | 0.917 | 49 | 0.101 | 13 | 51 | <0.0000001 |
|  | **EBA181** | -0.26 | 0.142 | 34 | 0.576 | 44 | 48 | 0.230 | 0.112 | 49 | 0.042 | 13 | 51 | <0.0000001 |
|  | **GLURP-R2** | -0.17 | 0.344 | 34 | 0.296 | 44 | 48 | 0.174 | 0.232 | 49 | 0.032 | 13 | 51 | <0.0000001 |
|  | **MSP2-ch150/9** | -0.27 | 0.117 | 34 | 0.028 | 44 | 48 | -0.121 | 0.409 | 49 | 0.812 | 13 | 51 | <0.0000001 |
|  | **MSP2-dd2** | -0.04 | 0.827 | 34 | 0.351 | 44 | 48 | -0.173 | 0.235 | 49 | 0.846 | 13 | 51 | <0.0000001 |
|  | **PfAMA1** | -0.51 | 0.002 | 34 | 0.391 | 44 | 48 | -0.017 | 0.906 | 49 | 0.476 | 13 | 51 | <0.0000001 |
|  | **PfMSP1-19** | -0.17 | 0.322 | 34 | 0.296 | 44 | 48 | -0.039 | 0.789 | 49 | 0.476 | 13 | 51 | 0.000161 |
|  | **RH22030** | -0.17 | 0.351 | 34 | 0.852 | 44 | 48 | 0.177 | 0.223 | 49 | 0.270 | 13 | 51 | <0.0000001 |
|  | **RH42** | -0.05 | 0.792 | 34 | 0.332 | 44 | 48 | -0.094 | 0.521 | 49 | 0.154 | 13 | 51 | <0.0000001 |
|  | **RH51** | -0.03 | 0.851 | 34 | 0.391 | 44 | 48 | 0.004 | 0.978 | 49 | 0.681 | 13 | 51 | 0.0000006 |
|  | **Pfs4845-10c** | -0.20 | 0.254 | 34 | 0.179 | 44 | 48 | -0.110 | 0.463 | 49 | 0.713 | 13 | 51 | 0.0043777 |
|  | **Pfs230-CMB** | 0.10 | 0.569 | 34 | 0.628 | 44 | 48 | -0.090 | 0.537 | 49 | 0.779 | 13 | 51 | 0.0000214 |

**Supplementary table 3: Infectivity data (DMFA) for individuals with co-incident gametocyte density measures.** IQR = Interquartile range.

|  | **Individuals tested in DMFA** | **Individuals infectious** | **Total DMFA assays performed** | **DMFA assays infectious** | **Mean number of assays performed per person**  Mean (range) | **Number of mosquitoes dissected per assay**  Mean (range) | **Percent mosquitoes infected/number of mosquitoes dissected (all individuals)**  % n/N | **Number of mosquitoes infected/number of mosquitoes dissected (infectious individuals)**  % n/N | **Average % mosquitoes infected by each infectious individual**  Median (IQR) |
| --- | --- | --- | --- | --- | --- | --- | --- | --- | --- |
| **Chronic** | 60/60 | 46.7% (28/60) | 160 | 25.6% (41/160) | 2.67 (1-3) | 80.0 (76.0-83.0) | 4.5% (554/12,405) | 17.9% (554/3089) | 7.6% (5.3-21.5) |
| **Incident** | 34/48 | 5.9% (2/34) | 52 | 3.8% (2/52) | 1.53 (1-3) | 64.5 (50.5-80.5) | 0.1% (5/3,403) | 3.5% (5/143) | 3.7 (2.5-4.8) |

**Supplementary table 4. Primer sequences for stage-specific parasite quantification.**

| **Target** | **Primer/probe** | **Sequence** |
| --- | --- | --- |
| *PfMGET* | PfMGET FW: | 5’- cggtccaaatataaaatcctg -3’ |
|  | PfMGET RV: | 5’- tgtgtaacgtatgattcattttc-3’ |
|  | PfMGET Probe: | 5’-FAM-cagctccagcattaaaaacac-BHQ1-3’ |
| *Pfs25* | Pfs25-FW: | 5’-GAAATCCCGTTTCATACGCTTG-3’ |
|  | Pfs25-RV: | 5’-AGTTTTAACAGGATTGCTTGTATCTAA-3’ |
| *18S* | Pf 18S FW: | 5’- GTAATTGGAATGATAGGAATTTACAAGGT-3’ |
|  | Pf 18S RV: | 5’- TCAACTACGAACGTTTTAACTGCAAC-3’ |
|  | Pf 18S probe: | 5’-6FAM-AACAATTGGAGGGCAAG–MGBNFQ-3’ |
| *pfap2-g* | AP2-g FW: | 5'- tggtggtaataagaacaacagaggt-3' |
|  | AP2-g RV: | 5'- ccatcataatcttcttcttcgtcg-3' |
| *sbp1* | SBP1 FW: | 5'- gcaaaacaagccgtacatgttg - 3' |
|  | SBP1 RV: | 5'- ttgctaggtaatatccttttctttttcc - 3' |
| *gexp-5* | PfGEXP05 FW: | 5'- gggctgttatgtatatttttattgaatttt- 3' |
|  | PfGEXP05 RV: | 5'- cattcgtttcattttcaccacttc- 3' |

1. Kastenmüller K, Espinosa DA, Trager L, et al. Full-Length *Plasmodium falciparum* Circumsporozoite Protein Administered with Long-Chain Poly(I·C) or the Toll-Like Receptor 4 Agonist Glucopyranosyl Lipid Adjuvant-Stable Emulsion Elicits Potent Antibody and CD4+ T Cell Immunity and Protection in Mice. Infection and Immunity **2013**; 81(3): 789-800.

2. Richards JS, Stanisic DI, Fowkes FJI, et al. Association between Naturally Acquired Antibodies to Erythrocyte-Binding Antigens of Plasmodium falciparum and Protection from Malaria and High-Density Parasitemia. Clin Infect Dis **2010**; 51(8): e50-e60.

3. Theisen M, Vuust J, Gottschau A, Jepsen S, Høgh B. Antigenicity and immunogenicity of recombinant glutamate-rich protein of Plasmodium falciparum expressed in Escherichia coli. Clin Diagn Lab Immunol **1995**; 2(1): 30-4.

4. Polley SD, Conway DJ, Cavanagh DR, et al. High levels of serum antibodies to merozoite surface protein 2 of Plasmodium falciparum are associated with reduced risk of clinical malaria in coastal Kenya. Vaccine **2006**; 24(19): 4233-46.

5. Taylor RR, Smith DB, Robinson VJ, McBride JS, Riley EM. Human antibody response to Plasmodium falciparum merozoite surface protein 2 is serogroup specific and predominantly of the immunoglobulin G3 subclass. Infection and immunity **1995**; 63(11): 4382-8.

6. Collins CR, Withers-Martinez C, Bentley GA, Batchelor AH, Thomas AW, Blackman MJ. Fine Mapping of an Epitope Recognized by an Invasion-inhibitory Monoclonal Antibody on the Malaria Vaccine Candidate Apical Membrane Antigen 1. J Biol Chem **2007**; 282(10): 7431-41.

7. Burghaus PA, Holder AA. Expression of the 19-kilodalton carboxy-terminal fragment of the Plasmodium falciparum merozoite surface protein-1 in Escherichia coli as a correctly folded protein. Mol Biochem Parasitol **1994**; 64(1): 165-9.

8. Triglia T, Thompson J, Caruana SR, Delorenzi M, Speed T, Cowman AF. Identification of proteins from Plasmodium falciparum that are homologous to reticulocyte binding proteins in Plasmodium vivax. Infect Immun **2001**; 69(2): 1084-92.

9. Reiling L, Richards JS, Fowkes FJ, et al. The Plasmodium falciparum erythrocyte invasion ligand Pfrh4 as a target of functional and protective human antibodies against malaria. PLoS One **2012**; 7(9): e45253.

10. Hjerrild KA, Jin J, Wright KE, et al. Production of full-length soluble Plasmodium falciparum RH5 protein vaccine using a Drosophila melanogaster Schneider 2 stable cell line system. Sci Rep **2016**; 6: 30357.

11. Singh SK, Roeffen W, Andersen G, et al. A Plasmodium falciparum 48/45 single epitope R0.6C subunit protein elicits high levels of transmission blocking antibodies. Vaccine **2015**; 33(16): 1981-6.

12. Farrance CE, Rhee A, Jones RM, et al. A Plant-Produced Pfs230 Vaccine Candidate Blocks Transmission of Plasmodium falciparum. Clinical and Vaccine Immunology **2011**; 18(8): 1351-7.

**References**
