# Supplementary material for: Higher gametocyte production and mosquito infectivity in chronic compared to incident Plasmodium falciparum infections
Source: Nat Commun. 2021 Apr 26;12:2443. doi: 10.1038/s41467-021-22573-7 (PMC8076179; doi:10.1038/s41467-021-22573-7)
Supplement: Supplementary file 3 — Reporting Summary [file 41467_2021_22573_MOESM3_ESM.pdf]

nature research

Corresponding author(s): Teun Bousema

Last updated by author(s): Feb 8, 2021

Reporting Summary

Nature Research wishes to improve the reproducibility of the work that we publish. This form provides structure for consistency and transparency in reporting. For further information on Nature Research policies, see Authors & Referees and the Editorial Policy Checklist .

Statistics

For all statistical analyses, confirm that the following items are present in the figure legend, table legend, main text, or Methods section.

n/a	Confirmed	
☐	☒	The exact sample size (*n*) for each experimental group/condition, given as a discrete number and unit of measurement
☐	☒	A statement on whether measurements were taken from distinct samples or whether the same sample was measured repeatedly
☐	☒	The statistical test(s) used AND whether they are one- or two-sided<br>*Only common tests should be described solely by name; describe more complex techniques in the Methods section.*
☐	☒	A description of all covariates tested
☐	☒	A description of any assumptions or corrections, such as tests of normality and adjustment for multiple comparisons
☐	☒	A full description of the statistical parameters including central tendency (e.g. means) or other basic estimates (e.g. regression coefficient) AND variation (e.g. standard deviation) or associated estimates of uncertainty (e.g. confidence intervals)
☐	☐	For null hypothesis testing, the test statistic (e.g. *F*, *t*, *r*) with confidence intervals, effect sizes, degrees of freedom and *P* value noted<br>*Give P values as exact values whenever suitable.*
☒	☐	For Bayesian analysis, information on the choice of priors and Markov chain Monte Carlo settings
☒	☐	For hierarchical and complex designs, identification of the appropriate level for tests and full reporting of outcomes
☐	☒	Estimates of effect sizes (e.g. Cohen's *d*, Pearson's *r*), indicating how they were calculated

Our web collection on statistics for biologists contains articles on many of the points above.

Software and code

Policy information about availability of computer code

Data collection	All data were recorded on paper forms, double entered in Microsoft Access 2010 and imported into Stata version 15.0.
Data analysis	Stata version 15.0

For manuscripts utilizing custom algorithms or software that are central to the research but not yet described in published literature, software must be made available to editors/reviewers. We strongly encourage code deposition in a community repository (e.g. GitHub). See the Nature Research guidelines for submitting code & software for further information.

Data

Policy information about availability of data

All manuscripts must include a data availability statement. This statement should provide the following information, where applicable:

- Accession codes, unique identifiers, or web links for publicly available datasets
- A list of figures that have associated raw data
- A description of any restrictions on data availability

Underlying datasets have been deposited in the Dryad repository (https://doi.org/10.5061/dryad.pc866t1n3)

Field-specific reporting

Please select the one below that is the best fit for your research. If you are not sure, read the appropriate sections before making your selection.

☒ Life sciences ☐ Behavioural & social sciences ☐ Ecological, evolutionary & environmental sciences

For a reference copy of the document with all sections, see nature.com/documents/nr-reporting-summary-flat.pdf

Life sciences study design

All studies must disclose on these points even when the disclosure is negative.

Sample size	Sample size is based on estimating the fraction of incident infections that develop mature gametocytes within two weeks after first detection of asexual parasites by molecular methods. To account for variability between subjects, we assume each subject has his own probability p that an infection leads to mature gametocytes (Bernoulli distribution). This p varies from subject to subject according to a distribution with mean mu=0.8 and a certain variance sigma2 (a beta-distribution). The fraction of all infections that leads to mature gametocytes is then asymptotically normally distributed with mean mu and variance se2= (mu*(1-mu) – sigma2) /(n) + sigma2/n, where n is the number of infections. Then the power that the lower limit of the 95% CI (of the fraction of infections that is MG positive) is above a limit L is 80% if (L-mu)/se <= 2.8. To detect whether ≥ 40% of infections develop MG within 2 weeks after first detection of infection by molecular methods with a lower limit of the 95% CI of 20%, 45 monitored infections are required. Given recent estimates of malaria incidence from the phase 3 RTS,S vaccine trial in areas with transmission intensity similar to our study area, we can conservatively assume that the incidence of infection is at least 0.8 infections/child-season. Acknowledging heterogeneity in malaria exposure and 20% lost to follow-up, we conservatively enroll 100 children in this cohort, expecting at least 45 monitored infection which is fourfold more than has previously been used to determine parasite dynamics in naturally infected individuals [49]. We also aim to intensively follow a similar number of chronic infections during the second phase of the study. If we do not manage to reach this number of infections, we will extend our data collection to a next transmission season and will submit an amendment for this to ethics committees. Our sample size with 13 observation time-points from 45 infections per longitudinal study will comprise a highly informative dataset to study the dynamics of gametocytes in naturally infected individuals. The first cohort resulted in 52 individuals enrolled and 51 children with a successfully monitored infection, including 37 that had infection detected before developing symptoms. This indicates that recruiting 400 children in the monthly monitoring is very likely to result in ≥50 monitored chronic infections.
Data exclusions	A total of 253 individuals were screened for participation in the incident infection sub-study; 80 were confirmed Plasmodium negative by nested PCR (nPCR), willing to participate, and met all other criteria for enrolment (table 1). Incident infections were detected by nPCR for 65% (52/80) of individuals after a median participation of 27 days (interquartile range [IQR] 20-41). After retrospective qPCR assays were performed on all pre- and post- enrolment samples, 4 individuals were excluded from subsequent analysis because they had parasites measured by qPCR more than 2 weeks before they were detected by nPCR (i.e. positivity >2 weeks prior to inclusion in the incident cohort was judged exclude them from catgorisation as an 'incident' infection), leaving 48 individuals in the incident infection cohort.
Replication	Two cohorts were defined with identical data collection over 2 seasons. The incident infection cohort from 2015 was repeated in independent infections in 2017 to assess consistency of findings. Similarly, the chronic infection cohort from 2016 was repeated in 2017.
Randomization	No randomization took place; we followed infections that were naturally acquired and therefore could not randomize children to the incident infection cohort and the chronic infection cohort.
Blinding	No formal blinding was performed since the nature of data collection made blinding impossible. In the years 2015 (incident infection cohort) and 2016 (chronic infection cohort), only one of the two cohorts was followed and staff involved in data collection in Burkina Faso was thus aware of the cohort study participants were in; in 2017 both cohorts ran simultaneously. All staff involved in laboratory assessments, including mosquito feeding assays, immunological assays and molecular assays were blinded to the symptomatic status of participants.

Reporting for specific materials, systems and methods

We require information from authors about some types of materials, experimental systems and methods used in many studies. Here, indicate whether each material, system or method listed is relevant to your study. If you are not sure if a list item applies to your research, read the appropriate section before selecting a response.

Materials & experimental systems

n/a	Involved in the study	
☐	☒	Antibodies
☒	☐	Eukaryotic cell lines
☒	☐	Palaeontology
☒	☐	Animals and other organisms
☐	☒	Human research participants
☒	☐	Clinical data

Methods

n/a	Involved in the study	
☒	☐	ChIP-seq
☒	☐	Flow cytometry
☒	☐	MRI-based neuroimaging

Antibodies

Antibodies used	Naturally acquired malaria antigen specific antibodies in the participants plasma were quantified using bead based assays as described in the methods. In short, malaria protein antigens (described in Supplemental table 1) were hybridised to Luminex beads, and probed with participant plasma. The probed beads were then incubated with a generic R-Phycoerythrin conjugated goat anti-human IgG (Jackson Immuno, PA, USA) at a dilution of 1:200.
Validation	No commercial primary antibodies were used in this study. The above-mentioned secondary antibody has a catalogues number 109-116-098.

Human research participants

Policy information about studies involving human research participants

Population characteristics	The median age in the incident cohort [n=48] was 7 [range 5-10], and 38% were female. The median age in the chronic cohort [n=60] was 8 [range 6-10], and 42% were female. General inclusion criteria are described below. In the incident infection cohort, aiming to characterize incident infection dynamics, individuals were cleared of existing infection and monitored weekly by PCR for up to 6 months to detect new infections at their onset. In the chronic cohort, aiming to characterize chronic asymptomatic infections, individuals with no clinical disease were enrolled for monthly monitoring, and were classified as having chronic and asymptomatic infection when parasites were detected on consecutive visits by PCR in the absence of symptoms.
Recruitment	Individuals included in this study were aged ≥5-10 years, were willing to provide repeated blood samples over a 6-month period, and if their caregivers provide informed consent. Exclusion criteria were complicated symptomatic malaria (defined according to standard World Health Organization criteria), anaemia (Hb<8g/dL), presence of any (chronic) illness that requires immediate clinical care, family history of sudden death or of congenital or clinical conditions known to prolong QTc interval (e.g. family history of symptomatic cardiac arrhythmias, clinically relevant bradycardia or severe cardiac disease), current treatment with drugs which could induce a lengthening of QT interval, known history of hypersensitivity, allergic or adverse reactions to piperaquine or other aminoquinolones, severe malnutrition (weight-for-height being below -3 standard deviation or less than 70% of median of the NCHS/WHO normalized reference values), weight below 15 kg, and current or previous participation in malaria vaccine trials.<br>Self-selection bias cannot be completely ruled out; parents whose children were more affected by malaria in preceding years might have been more willing to consent to enrollment. However, screening and initial consent started at the end of the dry-season when (recent) malaria exposure is considered negligible and unlikely to influence willingness to participate in the study.
Ethics oversight	The study protocol was approved by the ethical review boards of the London School of Hygiene and Tropical medicine (LSHTM) (#9008), the Centre National de Recherche et de Formation sur le Paludisme (CNRFP) (Deliberation numbers 2015-3-033) and the Burkina Faso National Ethical Committee for Health Research.

Note that full information on the approval of the study protocol must also be provided in the manuscript.
